# Upper Limb Immobilisation: A Neural Plasticity Model with Relevance to Poststroke Motor Rehabilitation

**DOI:** 10.1155/2016/8176217

**Published:** 2015-12-30

**Authors:** Leonardo Furlan, Adriana Bastos Conforto, Leonardo G. Cohen, Annette Sterr

**Affiliations:** ^1^School of Psychology, Faculty of Health and Medical Sciences, University of Surrey, Guildford GU2 7XH, UK; ^2^Neurology Clinical Division, Clinics Hospital, São Paulo University, Avenida Dr. Enéas C. Aguiar 255/5084, 05403-010 São Paulo, SP, Brazil; ^3^Instituto de Ensino e Pesquisa, Hospital Israelita Albert Einstein, Avenida Albert Einstein 627/701, 05601-901 São Paulo, SP, Brazil; ^4^Human Cortical Physiology and Stroke Rehabilitation Section, National Institutes of Neurological Disorders and Stroke, National Institutes of Health, Building 10, Room 7D54, Bethesda, MD 20892, USA

## Abstract

Advances in our understanding of the neural plasticity that occurs after hemiparetic stroke have contributed to the formulation of theories of poststroke motor recovery. These theories, in turn, have underpinned contemporary motor rehabilitation strategies for treating motor deficits after stroke, such as upper limb hemiparesis. However, a relative drawback has been that, in general, these strategies are most compatible with the recovery profiles of relatively high-functioning stroke survivors and therefore do not easily translate into benefit to those individuals sustaining low-functioning upper limb hemiparesis, who otherwise have poorer residual function. For these individuals, alternative motor rehabilitation strategies are currently needed. In this paper, we will review upper limb immobilisation studies that have been conducted with healthy adult humans and animals. Then, we will discuss how the findings from these studies could inspire the creation of a neural plasticity model that is likely to be of particular relevance to the context of motor rehabilitation after stroke. For instance, as will be elaborated, such model could contribute to the development of alternative motor rehabilitation strategies for treating poststroke upper limb hemiparesis. The implications of the findings from those immobilisation studies for contemporary motor rehabilitation strategies will also be discussed and perspectives for future research in this arena will be provided as well.

## 1. Introduction

The human brain changes itself in response to different types of experience through the reorganisation of its neuronal connections. This phenomenon is known as neural plasticity. It is suggested that it involves firstly a short-term modulation in the strength of existing pathways and that, over time, with prolonged exposure, such modulation might be followed by more stable, longer-term structural changes in brain networks [1]. Neural plasticity manifests itself during brain development [2], motor and perceptual skill learning [3, 4], and also during/after central nervous system (CNS) diseases/disorders [5], to name a few. The reorganisation of neuronal connections in the brain within these and also other contexts is commonly seen as being beneficial to the individual. However, neural plasticity can also be detrimental [6]. When brain changes are associated with improvements in the individual's behavioural capacity, neural plasticity is referred to as being adaptive [7–9]. On the other hand, when brain changes are linked to behavioural deterioration, or adverse consequences to the individual, neural plasticity is referred to as being maladaptive [10, 11].

Thus, it follows from the above that, by identifying neural plasticity and its behavioural correlates, together with an understanding of its mechanisms and likely causal factors, one can develop strategies to enhance adaptive and/or suppress maladaptive brain changes in order to improve the individual's behavioural capacity [12]. This opportunity for intervention is of paramount importance to the clinical context of CNS diseases/disorders, where many different patterns of neural plasticity have been identified, each of which being associated with either positive or negative behavioural outcomes [5]. This is particularly true for stroke [5].

Stroke is a major cause of acquired physical disability in adults worldwide. Motor deficits affecting the upper limb are a common manifestation of stroke and greatly contribute to decreasing the individual's functional performance and thereby to the level of disability that is achieved [13]. It is widely appreciated that motor rehabilitation after stroke plays an essential role in reducing the individual's physical disability [14–16]. This paper will focus on the motor rehabilitation of the paretic upper limb after stroke.

Until about the late 1980s, neurorehabilitation professionals, despite recognising the importance of motor rehabilitation, had a somewhat restricted therapeutic armamentarium for treating stroke-related motor deficits, such as upper limb hemiparesis [17]. This was in part due to our relatively limited understanding of the neural mechanisms underlying motor deficits/recovery after hemiparetic stroke at that time [18]. By then, neurorehabilitation consisted mostly in teaching patients compensatory behaviours with their preserved body functions and/or in the utilisation of so-called “neurophysiological approaches” that lacked a strong scientific basis and had their therapeutic efficacy questioned [19]. Fortunately, from that time onwards, with the advent of noninvasive neurophysiological and neuroimaging tools for assessing/altering brain activity/function in humans, and the increasingly greater utilisation of such tools in stroke patients, together with major advances in the development and use of animal models of stroke, the scenario began to change. For instance, over the past twenty five years or so, the findings from numerous correlational and experimental studies conducted with brain injured adult humans and animals have substantially enlightened our understanding of the neural plasticity that occurs after hemiparetic stroke [5, 20–26]. Overall, this progress has contributed to the formulation of “theories” of motor recovery after stroke. In short, these theories identify neural plasticity patterns, both adaptive and maladaptive, and delineate their mechanisms and likely causal factors, for example, damage to, or activity changes in, particular brain regions or pathways and the presence or absence of specific behavioural or neural signals, to name a few. Such mechanistic understanding of poststroke motor deficits/recovery has, in turn, allowed for the theoretical conceptualisation and subsequent development of new, science-based motor rehabilitation strategies to treat upper limb hemiparesis, most of which are still under investigation [27–30]. In parallel, other strategies that were being conceptualised and developed from otherwise different, yet complementary scientific perspectives, for example, behavioural psychology and multidisciplinary movement science, have found in those neural plasticity-based theories of motor recovery a strong neuroscientific support. This has further contributed to the establishment of these latter strategies as treatment options for poststroke upper limb hemiparesis [31–34]. In this paper, we will refer to both these and those previously mentioned newly developed strategies as “contemporary motor rehabilitation strategies.” Altogether, this has not only represented a major revolution in neurorehabilitation but also nurtured a more optimistic prospect to the field.

### 1.1. The Problem

Notwithstanding the aforementioned achievements, several challenges have yet to be addressed in the arena of poststroke motor rehabilitation research, and this has consequences to clinical practice worldwide [35–38]. One of these challenges, for instance, is that, in general, contemporary motor rehabilitation strategies for poststroke upper limb hemiparesis are most compatible with the recovery profiles of relatively high-functioning stroke survivors and therefore do not easily translate into benefit to individuals with poorer residual function. This is often in contrast to the day-to-day scenario of real-world rehabilitation settings, where many of the patients in need for motor rehabilitation after a stroke are usually at closer proximity to the lower end of the spectrum of functional recovery. This discrepancy may stem from the fact that most of the investigations performed so far in this field, particularly the studies testing motor rehabilitation strategies, have, for several reasons, focused primarily on well recovered stroke models [39] (but see also [40]). As a consequence, many stroke survivors, such as those sustaining low-functioning upper limb hemiparesis, remain with rather limited rehabilitation options [41].

Therefore, in light of the above, we argue that there is a need for alternative motor rehabilitation strategies for stroke, if clinical practice demands are to be met more widely and effectively. Essentially, these alternative strategies have to fulfill at least two requirements. First, they should be able to promote the adaptive neural plasticity pattern(s) currently identified as instrumental to poststroke motor recovery. Second, and most critically, their ability to do that must not be influenced by the individual's level of residual function. Importantly, such strategies are likely not only to be more compatible with the recovery profiles of stroke survivors with poor residual function, but also to translate into benefit to the entire spectrum of functional recovery. However, in order for this to be achieved, models that more closely resemble a condition of low-functioning upper limb hemiparesis are required. As will be elaborated in the remainder of this paper, upper limb immobilisation is a key candidate for this position.

Below, we will first briefly describe two prevailing theories of motor recovery after stroke and some of the contemporary motor rehabilitation strategies for treating poststroke upper limb hemiparesis that have been largely underpinned by these theories. This will be followed by an overview of upper limb immobilisation studies in healthy adult humans and animals and a discussion as to how the findings from these studies could inspire the creation of a model that, we believe, is likely to be of particular relevance to the context of motor rehabilitation after stroke. Our premise here is that an upper limb immobilisation model, by capitalising on both, current theories of motor recovery after stroke and the shortage of physical or overt movements, which is a hallmark of low-functioning hemiparesis, offers a compelling neurobehavioural framework upon which alternative motor rehabilitation strategies for treating upper limb hemiparesis can be envisioned, firstly developed and tested in healthy individuals, and then ultimately translated into the clinical context of poststroke motor rehabilitation.

## 2. Theories of Motor Recovery after Stroke

### 2.1. Background Information: Cortical Motor Representations, What They Reflect, and What Drives Their Organisation

The motor cortex contains representations of body parts [42, 43]. Throughout this paper, we will refer to such representations as “cortical motor representations.” These representations are commonly derived through electrical stimulation of the precentral cortex in the frontal lobe, with either noninvasive or invasive techniques, such as transcranial magnetic stimulation (TMS) and intracortical microstimulation (ICMS), respectively. While TMS is more frequently used with humans, ICMS is typically employed in animal research. The mechanism of action of these two techniques, when they are used for that purpose, usually involves the stimulation of axons from local intracortical circuits that synapse onto corticospinal neurons that then synapse onto contralateral spinal motor neurons innervating skeletal muscles, which, in turn, act upon specific body parts. However, direct activation of corticospinal neurons may also occur under certain conditions with both TMS and ICMS. The response to stimulation is then recorded in the periphery visually, that is, through the visualisation of movement of the corresponding body part(s), and/or by means of surface electromyography of the involved muscle(s). Thus, when derived, cortical motor representations may be considered as being a measure of both the amount of cortical/corticospinal tissue that is being dedicated to the motor control of a particular body part, which can be inferred from the size of the obtained representation over the cortex or scalp, and the strength or efficacy of this control at the time of stimulation, which can be inferred from the intensity of the recorded response in the periphery. The latter, in turn, usually indicates the excitability of the cortical/corticospinal components of the representation that are activated by the stimulation. As Phillips and Porter (1977) commented on the use of electrical stimulation for deriving cortical motor representations, “This leaves us free to concentrate on its merits as a tool for mapping the outputs that are available for selection by the intracortical activities that it cannot itself evoke” (Phillips and Porter 1977, p. 37) ([44], p. 304). In other words, the size and/or excitability of a cortical motor representation corresponding to a particular body part can be thought of as reflecting the individual's motor capacity/skill with that body part [45].

Contrary to what was once held, cortical motor representations are by no means static entities. Instead, numerous neurophysiological studies performed with TMS and ICMS on adult humans and animals in the past years have consistently demonstrated that such representations are rather flexible or dynamic and that one fundamental driver of their organisation, in terms of both their size and excitability, is the amount of use or sensorimotor experience with the corresponding body part(s). In general, conditions of increased use or sensorimotor experience that increase activity in the efferent and/or afferent neural signalling pathways targeting and/or coming from a particular body part, or parts, for example, motor skill learning/acquisition and somatosensory stimulation, induce an increase in the size and/or excitability of the cortical motor representation(s) of the involved body part(s). This is often accompanied by gains in motor capacity with the involved body part(s) and therefore reflects adaptive neural plasticity. Conversely, conditions of decreased use or sensorimotor experience that decrease or even cease activity in those pathways, for example, brain or peripheral nerve lesions, amputation, spinal cord injury, and ischemic nerve block-mediated local anesthesia, lead to a decrease in the size and/or excitability of the cortical motor representation(s) of the affected body part(s). This usually parallels a reduction in motor capacity with the affected body part(s) and therefore reflects maladaptive neural plasticity [46–49]. There is evidence that these changes in the size and/or excitability of cortical motor representations, both adaptive and maladaptive, are mediated by, among other factors, synaptic strength modification processes, such as long-term potentiation (LTP) and long-term depression (LTD), occurring within intracortical circuits in the motor cortex (see [45, 49] for further details).

### 2.2. Theories of Poststroke Motor Recovery

Findings collated from several neurophysiological and neuroimaging investigations performed with brain injured adult humans and animals have contributed so far to the formulation of at least two complementary theories of motor recovery after hemiparetic stroke. In the following sections, we will refer to these theories as the “*reactivation*” and “*rebalancing*” theories. It might be worth mentioning that what we call theories here has actually appeared more frequently in the literature as “concepts,” “models,” or “neural strategies” for motor recovery, rather than as theories per se. Also, other names rather than “reactivation” and “rebalancing” have been more commonly used. Nevertheless, the underlying principles/mechanisms have been fully preserved and the terms employed here were chosen simply for the purposes of this paper.

The “*reactivation*” theory makes three main assumptions. First, in the healthy brain, increased use or sensorimotor experience in the form of motor skill acquisition promotes adaptive neural plasticity, that is, increases in the size and/or excitability, of the cortical motor representation(s) of the involved body part(s). Second, motor deficits after hemiparetic stroke are due not only to the structural lesion itself, but, critically, also to maladaptive neural plasticity occurring in structurally intact, residual brain areas connected to the damaged region(s). Of special interest here is the adjacent, perilesional tissue surrounding the stroke core. After the stroke, this region may still contain some residual cortical/corticospinal components of the cortical motor representations corresponding to the paretic body parts. When this is the case, it follows that, over time, these spared representations often undergo a substantial reduction in their size and/or excitability. This condition of perilesional depression is usually the combined result of phenomena that are initially triggered by the stroke lesion, such as diaschisis and learned nonuse, and that are subsequently aggravated by a state of substantially reduced usage of, and hence reduced sensorimotor experience with, the paretic body parts. Third, the mechanisms of the above can interact so that after hemiparetic stroke, increased use or sensorimotor experience with the paretic body parts in the form of motor skill (re)acquisition may adaptively modulate perilesional neural plasticity. Therefore, this theory predicts that, by increasing use of the paretic body parts in a skill (re)acquisition-like manner, the cortical motor representations corresponding to these body parts, which may still have some residual components available in the perilesional tissue, are* reactivated* and adaptively stimulated; that is, they increase their size and/or excitability and thereby improve the individual's motor function [50, 51] (see also [20, 52, 53] for further discussion).

**Figure 1:**
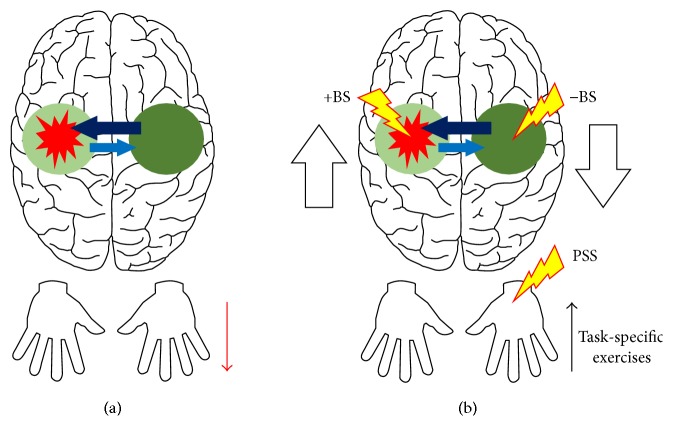
This figure illustrates the interaction between two prevailing theories of motor recovery after stroke, named here as the “reactivation” and “rebalancing” theories (a), and some of the contemporary motor rehabilitation strategies for treating poststroke upper limb hemiparesis that have been largely underpinned by these theories (b).* Red explosion-like balloon*: hemiparetic stroke.* Light green circle*: depression, that is, decreased size and/or excitability, of residual cortical motor representations in the adjacent, perilesional tissue.* Dark green circle*: overactivity of homologous cortical motor representations in the opposite, undamaged cerebral hemisphere.* Light blue arrow*: decreased transcallosal inhibition.* Dark blue arrow*: increased transcallosal inhibition.* Red thin downward arrow*: reduced use of the paretic upper limb contralateral to the stroke side.* Black thin upward arrow*: increased skilled use of the paretic upper limb through physiotherapy in the form of task-specific exercises.* Red-yellow bolts*: adjunctive therapies, such as excitatory and inhibitory brain stimulation (+BS and −BS, resp.) and peripheral somatosensory stimulation (PSS), to be combined with physiotherapy exercises.* White tick upward arrow*: increase activity in the ipsilesional motor cortex.* White tick downward arrow*: decrease activity in the contralesional motor cortex. See text for further details.

The “*rebalancing*” theory, on the other hand, suggests that motor deficits after hemiparetic stroke result, apart from the structural lesion itself, from an interaction between depression of the perilesional tissue and further maladaptive neural plasticity involving both the ipsilesional and the contralesional cerebral hemisphere. For instance, a hemiparetic stroke, besides selectively disrupting lateralized motor control networks, often compromises transcallosal circuits that regulate the interhemispheric interactions between cortical areas. Of particular relevance here are the inhibitory transcallosal circuits that connect the cortical motor representations in the motor cortex of one hemisphere to those in the motor cortex of the contralateral hemisphere. The result is a reduction in the inhibition from the motor cortex of the stroke-affected hemisphere to the homologous area in the opposite, unaffected hemisphere. Such disinhibition usually leads to an abnormally increased activity in the contralesional motor cortex, which, in turn, causes excessive transcallosal inhibition from this area towards the homologous region in the ipsilesional hemisphere. This phenomenon is likely to be aggravated by a concomitant compensatory increased use of the less-affected body side, which contributes to further increasing activity in the contralesional motor cortex. Overall, it is suggested that such abnormal interhemispheric inhibition would be superimposed to an already existing condition of reduced perilesional activation in the affected hemisphere and that this could, in turn, contribute to a further decrease in the size and/or excitability of potentially available residual cortical motor representations in that hemisphere. Accordingly, this theory predicts that motor recovery after hemiparetic stroke is facilitated/enhanced if the interhemispheric interactions between the cortical motor representations of the two homologous motor cortices are* rebalanced* and that this can be achieved by increasing activity in the ipsilesional and/or decreasing activity in the contralesional motor cortex [54–56] (see also [20, 21, 57] for further discussion). The two theories of motor recovery are illustrated in Figure 1(a).

## 3. Contemporary Motor Rehabilitation Strategies for Poststroke Upper Limb Hemiparesis

Overall, those two aforementioned theories of motor recovery currently form the neuroscientific basis of contemporary motor rehabilitation strategies for treating upper limb hemiparesis after stroke. These strategies might be broadly grouped into two complementary categories of interventions. The first category is primarily supported by the “*reactivation*” theory and currently constitutes the central pillar of modern neurorehabilitation. This category aims to increase skilled use of the paretic upper limb in order to* reactivate* and adaptively stimulate, that is, increase the size and/or excitability of, likely latent cortical motor representations in the damaged cerebral hemisphere. It consists mostly of physiotherapy exercises that are performed with the paretic upper limb and that are delivered in the form of repetitive and increasingly challenging task-specific exercises directed towards the (re)acquisition of motor skills [58, 59]. The second category of interventions, on the other hand, is based on both theories of motor recovery, but particularly on the “*rebalancing*” theory. This category aims to boost the effects of physiotherapy exercises through the use of adjunctive therapies that have the potential to* rebalance* the interhemispheric interactions between the two homologous motor cortices and that can be delivered in combination with task-specific practice. These adjunctive therapies include, but are not limited to, excitatory and inhibitory brain stimulation (+BS and −BS, resp.) [60–70] and peripheral somatosensory stimulation (PSS) [71–75]. The standard approach here is to deliver +BS to the ipsilesional motor cortex to increase its activity and/or −BS to the contralesional motor cortex to decrease its activity. In the case of PSS, the stimuli, which typically consist of low-intensity electric currents, are delivered transcutaneously to the paretic body part(s) in order to increase activity of the contralateral, ipsilesional motor cortex. These adjunctive therapies can be used either separately or in combination with each other. For instance, task-specific exercises can be coupled with both BS and PSS in order to further potentiate practice-induced adaptive neural plasticity (e.g., see [76]). Despite their promise, it is worth noting that these, and also other adjunctive therapies, are still under investigation and therefore are not yet widely used in clinical practice. The two categories of interventions are illustrated in Figure 1(b).

As can be noted, contemporary motor rehabilitation strategies for treating poststroke upper limb hemiparesis are centered in the physical/overt practice of motor tasks. Individuals are required to have a level of residual function that will ultimately allow them not only to actively engage with repetitive task practice, but also to perform increasingly difficult exercises [58, 59]. Therefore, while these rehabilitation strategies may undoubtedly translate into benefit for some stroke survivors, their overreliance on the availability of relatively high levels of residual function to overcome maladaptive and/or promote adaptive neural plasticity, that is, to promote motor recovery, represents a major obstacle. This is particularly true for individuals sustaining low-functioning upper limb hemiparesis who, due to poor residual function, cannot effectively engage with the physical practice of motor tasks in the way that is needed to promote the adaptive neural plasticity driving functional improvements. For these individuals, overt practice-based rehabilitation strategies are of very limited value. At this point, it is worth clarifying that a condition of poor residual function after a hemiparetic stroke, that is, low-functioning hemiparesis, at least in the way this concept is used in this paper, does not necessarily imply a severe neurological lesion completely destroying the cortical and/or corticospinal components of the cortical motor representations corresponding to the paretic body parts. While it stands to reason that such a severe lesion would result in poor residual function, the occurrence of the former is not a sine qua non for the presence of the latter. This is because many factors can influence/determine the level of residual function that is expressed by an individual after a hemiparetic stroke. For instance, it is well recognised that patients often experience substantial cardiorespiratory and skeletal muscle deconditioning after a hemiparetic stroke [77, 78]. As we have discussed elsewhere (e.g., see [79]), such deconditioning status, in turn, might greatly contribute to increasing their fatigue levels. Altogether, this can critically decrease the individual's physical capacity and ability to actively engage with the repetitive overt practice of progressive motor tasks, hence contributing to a low-functioning profile. Moreover, as already mentioned in the previous section, the motor deficits and hence the level of residual function that is shown by an individual after a hemiparetic stroke are currently thought to be largely influenced by maladaptive neural plasticity patterns occurring in structurally intact, residual brain areas/networks that were otherwise spared by the lesion. Therefore, a more holistic view here would be that a condition of low-functioning hemiparesis after stroke likely results from a complex interaction among different compromised body systems, instead of simply from the more direct effects of the neural damage, that is, the selective destruction of motor control pathways in the brain.

Thus, as suggested earlier in this paper, alternative motor rehabilitation strategies for stroke are currently needed. Essentially, in order to overcome the present obstacle, these alternative strategies must not rely on the individual's level of residual function to move from a state of maladaptive to one of adaptive neural plasticity, which may eventually translate into improved motor function. As will be discussed henceforth, an upper limb immobilisation model might be an attractive framework for developing such strategies.

## 4. Upper Limb Immobilisation and Neural Plasticity: What Do Human and Animal Studies Tell Us?

Given the fundamental role of use or sensorimotor experience in shaping cortical motor representations and thereby the individual's motor capacity/skill with the corresponding body part(s) (see Section 2.1), studies have started to investigate upper limb immobilisation as a paradigm of disuse or sensorimotor deprivation. In such studies, the entire upper limb, or part of it, is prevented to move by means of a bandage, splint, cast, and/or sling, either because of trauma or simply for experimental purposes. Particularly, these investigations have focused not only on the neural but also on the behavioural effects of immobilisation.

For example, in a classical study, Huber et al. (2006) immobilised the left arm and hand of healthy participants for 12 consecutive hours to explore the effects of sensorimotor deprivation on sensorimotor cortex activity, motor performance, and sleep slow wave activity (SWA). After the immobilisation, the authors found a substantial decrease in neuronal activity in the hand representation of the right sensory and motor cortices, as revealed by reduced somatosensory and motor potentials evoked through peripheral nerve stimulation and TMS, respectively. At the same time, the motor performance of the immobilised arm and hand had deteriorated, as indicated by an increase in hand-path area variability while individuals were reaching toward targets placed in front of them. Also, after immobilisation, there was a localised decrease in sleep SWA over the right sensorimotor cortex, which was detected through electroencephalography during subsequent sleep. Of note, a positive correlation was found between the changes in motor performance and the changes in both somatosensory evoked potentials and sleep SWA [80]. Subsequent studies have revealed comparable findings regarding the behavioural effects of immobilisation. In one of these studies, healthy participants had their upper limb immobilised for either 6 or 12 consecutive hours. It was found that after 12 but not 6 hours of immobilisation, motor control of the restricted limb was impaired, which was expressed through abnormalities in both hand trajectories and inter-joint coordination during reaching movements [81]. Similarly, in a different study, healthy individuals displayed altered motor performance in a reach-to-grasp task after 10 hours of continuous arm and hand immobilisation. Here, the transport phase of the reach-to-grasp movement was affected, in a way that reaching was slower and its peak velocity time was achieved earlier. An interesting finding from this study was that motor performance on the reach-to-grasp task quickly returned to baseline levels with only a few trials of practice after the immobilisation had been removed [82].

By employing the same immobilisation protocol from the latter study above, Avanzino et al. (2011) explored with TMS the effect of upper limb disuse on the interhemispheric interactions between the two homologous motor cortices. Additionally, they investigated whether this effect was modulated by the amount of use of the nonimmobilised limb. The study consisted of two groups. In one group, participants received no instructions regarding the amount of use of the nonimmobilised arm and hand, “free to move” group, whereas, in the other group, volunteers were instructed to limit contralateral movements, “limited movement” group. After the 10 consecutive hours of right arm and hand immobilisation, both groups showed decreased excitability of the hand representation in the left motor cortex and reduced interhemispheric inhibition from the left to the right hand cortical motor representation, with the latter effect being more pronounced in the “free to move” group. Of note, the excitability of the hand representation in the right motor cortex, as well as the interhemispheric inhibition from the right to the left hand cortical motor representation, increased only in the group that was free to move the left, nonimmobilised arm and hand [83].

In keeping with these latter findings, a longitudinal neuroimaging study by Langer et al. (2012) reported bilateral structural changes in the sensorimotor cortex and corticospinal tract of immobilised individuals recovering from upper limb fractures. After an average of 16 days of right arm and hand immobilisation, cortical thickness and fractional anisotropy (FA) were reduced in the hand representation of the left sensory and motor cortices and over the left corticospinal tract, respectively. In addition, motor abilities with the left arm and hand improved throughout the restriction period, presumably as a consequence of an increased use in order to compensate for the immobilisation of the contralateral limb. Interestingly, this behavioural change was associated with an increase in both cortical thickness and FA of the right motor cortex and corticospinal tract, respectively, and with a decrease in cortical thickness over the left sensorimotor cortex [84].

In another longitudinal study, in this case conducted with healthy adult monkeys, Milliken and colleagues (2013) investigated the effect of distal forelimb immobilisation on the somatotopic organisation of the corresponding cortical motor representation. Here, immobilisation periods varied from 38 to 248 days. Detailed cortical motor representations of the distal forelimb of the animals were obtained through ICMS techniques before, during, and after the immobilisation intervals. The authors found a progressive decrease in the size of the representation of the digits together with an equivalent increase in the size of the representation of more proximal limb parts, such as wrist and forearm. These changes were paralleled by a reduction in general/skilled use of the digits, which, in turn, was followed by a concomitant increase in the use of more proximal limb parts. In general, those cortical changes were reversed to baseline levels after removal of the immobilisation during a period of behavioural recovery, when the animals regained general/skilled use of the digits, either spontaneously or through forced use [85].

Complementing the above findings, Rosenkranz et al. (2014), by recording a variety of TMS-derived measures, recently showed that upper limb immobilisation, besides decreasing the excitability and/or size of the corresponding cortical motor representations in the contralateral motor cortex, also selectively alters neural plasticity and intracortical inhibition mechanisms within the same representations. Specifically, the authors showed that, after 8 consecutive hours of left hand immobilisation, individuals displayed increased responsiveness to paired associative stimulation (PAS) protocols, which are thought to induce long-term potentiation- (LTP-) like and long-term depression- (LTD-) like processes. In other words, both LTP and LTD were facilitated in the hand representation of the contralateral right motor cortex after immobilisation, presumably reflecting homeostatic adjustments that operate to increase the sensitivity of corticospinal neurons to available synaptic inputs and thereby prevent too much of a decrease in motor output capacity. In addition, after immobilisation, short-interval intracortical inhibition (SICI) was reduced in the hand representation of the right motor cortex, and this change was negatively correlated with the changes in excitability over this cortical area. Again, this is likely to reflect compensatory adjustments that act during/after immobilisation to maintain overall motor cortex excitability within a certain range. Finally, a correlation was also found between the reduction in the excitability of the hand representation over the right motor cortex and the strength of the effect of the PAS protocols. This correlation was positive for the LTP-inducing and negative for the LTD-inducing protocol [86]. The findings from this study, particularly those regarding the changes in neural plasticity and intracortical inhibition mechanisms, might explain, at least in part, the somatotopic reorganisation of cortical motor representations that usually occurs with upper limb immobilisation [86], such as that observed in the monkeys from the study mentioned previously [85].

Interestingly, the neural and behavioural effects that have been reported in immobilisation studies are, to a large extent, similar to those effects reported in investigations employing ischemic nerve block-mediated local anaesthesia, both in healthy individuals and after stroke [87–90]. This similarity emphasises the role of the presence/level of activity in the afferent and efferent neural signalling pathways associated with a particular body part in determining the size and/or excitability of its representation in the motor cortex and thereby the individual's motor capacity/skill with it. This observation, in turn, further corroborates the “*reactivation*” theory that was described previously in this paper (see Section 2.2).

In summary, the studies reviewed in this section provide evidence that upper limb immobilisation

- impairs motor performance with the restricted body part(s), even after short periods of restriction ranging from 10 to 12 hours, an effect that is largely reversible;
- induces maladaptive neural plasticity of the cortical motor representation(s) of the immobilised body part(s), which expresses itself in the form of motor behaviour deterioration, and which may vary from a simple reduction in the size and/or excitability of the involved representation(s) to structural changes in the cortical and corticospinal components of the same representation(s), depending on the duration of the immobilisation;
- affects the interhemispheric balance between the two homologous motor cortices in favour of the cortical motor representation(s) from the cerebral hemisphere ipsilateral to the immobilised body part(s), an effect that seems to be largely modulated by the amount of use of the contralateral, nonimmobilised limb;
- modulates neural plasticity and intracortical inhibition mechanisms within the cortical motor representation(s) of the restricted body part(s), a process which is likely to be responsible for the maladaptive neural plasticity of the involved representation(s). More specifically, such modulation occurs in a direction that appears to be predicted by the amount of depression that is induced by the restriction in the involved representation(s); for example, the larger the decrease in excitability, the greater the sensitivity of the representation(s) to subsequent LTP-like processes (with the opposite being true for LTD-like processes) and the smaller the reduction in SICI within the same representation(s).

## 5. Upper Limb Immobilisation as a Model for Developing Alternative Motor Rehabilitation Strategies for Poststroke Upper Limb Hemiparesis

**Figure 2:**
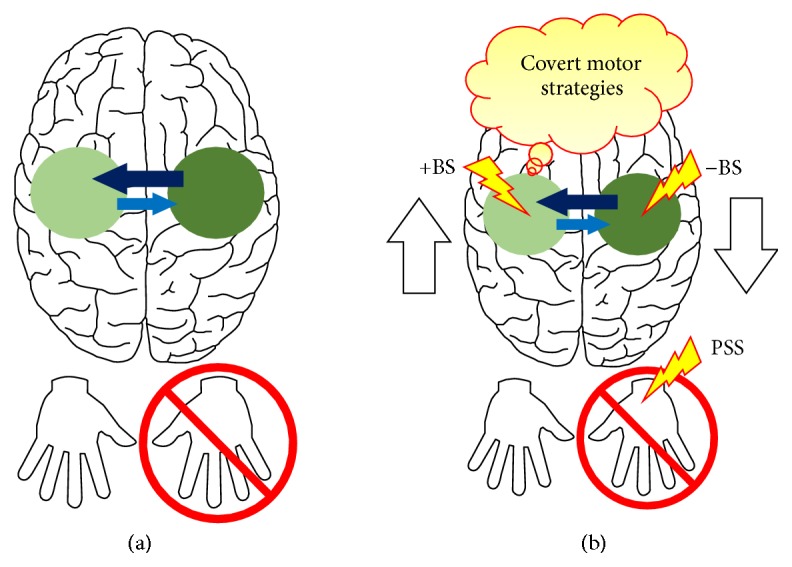
(a) highlights the similarity between the maladaptive neural plasticity patterns that often occur after a hemiparetic stroke and that are currently thought to play an important role in mediating the individual's motor deficits (see Figure 1(a) for comparison) and the neural plasticity patterns that are induced by upper limb immobilisation (*red crossed circle*) in otherwise healthy individuals.* Light green circle*: depression, that is, decreased size and/or excitability, of the cortical motor representation(s) corresponding to the immobilised body part(s).* Dark green circle*: overactivity of the homologous cortical motor representation(s) in the opposite cerebral hemisphere.* Light blue arrow*: decreased transcallosal inhibition.* Dark blue arrow*: increased transcallosal inhibition. (b) indicates potential interventions that could be delivered during a paradigm of upper limb immobilisation in healthy individuals in order to prevent maladaptive or promote adaptive neural plasticity in the motor system. These interventions might include, for instance, covert motor strategies, such as action observation (AO) and (likely) motor imagery (MI) (*red-yellow balloon*), and adjunctive therapies, such as excitatory and inhibitory brain stimulation (+BS and −BS, resp.) and peripheral somatosensory stimulation (PSS) (*red-yellow bolts*).* White tick upward arrow*: increase activity in the motor cortex contralateral to the immobilisation.* White tick downward arrow*: decrease activity in the motor cortex ipsilateral to the immobilisation. The idea here is that this might contribute to the development of alternative motor rehabilitation strategies for treating poststroke upper limb hemiparesis. See text for further details.

The proposal presented in this paper is that an upper limb immobilisation model offers a compelling neurobehavioural framework for developing alternative motor rehabilitation strategies for treating upper limb hemiparesis after stroke. As summarized previously, the neural effects of upper limb immobilisation are, to a large extent, similar to the maladaptive neural plasticity patterns that are often seen after hemiparetic stroke and that underpin current theories of motor recovery. To briefly recall, those effects involve both a reduction in the size and/or excitability of the cortical motor representation(s) corresponding to the immobilised body part(s) and changes in interhemispheric balance, with increased activity biased towards the cortical motor representation(s) from the cerebral hemisphere ipsilateral to the restricted limb. Indeed, in terms of maladaptive neural plasticity patterns, an upper limb immobilisation model largely resembles a hemiparetic stroke model, except of course for the absence of a true lesion (Figure 2(a)).

But if this is to be true, then what could be the advantages of using an upper limb immobilisation model as a stroke-like model for developing alternative motor rehabilitation strategies for upper limb hemiparesis, in place of currently used models (see Section 1.1)? Here, we argue that, besides inducing the maladaptive neural plasticity patterns that usually occur after a hemiparetic stroke and that are currently thought to play an important role in mediating the individual's motor deficits, the upper limb immobilisation model essentially promotes a condition of rather decreased, if any, overt use or sensorimotor experience, which in fact characterizes the restriction paradigms themselves. Such condition, in turn, is much more compatible with the recovery profiles of individuals with poor residual function. Thus, an upper limb immobilisation-based stroke-like model, in comparison to current models, more closely resembles a condition of low-functioning upper limb hemiparesis.

Importantly, within this context, if during a paradigm of upper limb immobilisation in healthy individuals interventions could be delivered in order to prevent the maladaptive neural plasticity that is induced by movement restriction, because these interventions would necessarily have to bypass overt movement execution, they could translate into alternative motor rehabilitation strategies for poststroke upper limb hemiparesis with a greater potential for benefiting those with poor residual function. Here, it is at least theoretically plausible that such interventions would be able to promote, for example, in low-functioning stroke survivors, the adaptive neural plasticity patterns currently identified as instrumental to poststroke motor recovery (see the “*reactivation*” and “*rebalancing*” theories in Section 2.2). Furthermore, the behavioural effects of immobilisation would also be of value to this context. For instance, it would be the case that interventions under investigation could aim to prevent not only the induction of maladaptive neural plasticity but also the deterioration of overt motor performance, which has also been a reported effect of immobilisation early after its removal (see beginning of Section 4).

### 5.1. Preventing Maladaptive/Promoting Adaptive Neural Plasticity during Upper Limb Immobilisation

Besides focusing on the neural and behavioural effects of upper limb immobilisation, recent studies have also started to explore how it can be used as a model of maladaptive neural plasticity upon which it is possible to test interventions that aim to prevent this neural plasticity from occurring. Specifically, researchers have employed upper limb immobilisation firstly to induce a depression, that is, a decrease in the size and/or excitability, of the cortical motor representation(s) of the restricted body part(s) and/or an interhemispheric imbalance between homologous representations in favour of the representation(s) ipsilateral to the immobilised body part(s). Then, they would try to prevent these changes from occurring through the concomitant delivery of interventions that are thought to have the potential to activate and adaptively stimulate cortical motor representations in the absence of overt use or sensorimotor experience with the corresponding body part(s). Below, we will review these studies and interventions within the context of motor rehabilitation after stroke.

#### 5.1.1. Background Information: Covert Motor Strategies and Their Infusion into Poststroke Motor Rehabilitation

Covert motor strategies might also be referred to as cognitive motor strategies. They include, for example, motor imagery (MI) and action observation (AO). These strategies are referred to as “covert motor” strategies because of their intrinsic ability to activate the motor system of the brain without the overt execution of movements. As suggested by Sharma et al. (2006), they may achieve that through the “backdoor” of the brain's motor system [91].

Theoretical support for MI and AO as covert motor strategies comes mostly from the “Simulation Theory,” which was proposed by Jeannerod almost fifteen years ago. According to this theory, the brain's motor system, including its cortical motor representations in the motor cortex, is part of a simulation network that is activated not only when we move, but also when we imagine ourselves or observe others moving. In this vein, the theory proposes that the neural substrate that is activated for the overt execution of a movement or action is, to a large extent, also activated by the imagination or observation of that same movement or action [92]. Studies from our group and several others performed with healthy participants have provided strong empirical support for the “Simulation Theory,” by showing an extensive overlap in neural activation between conditions of overt execution and conditions of imagination or observation of movements, and this includes activation of the cortical motor representation(s) of the involved body part(s) [93–98]. In addition, we have also shown that this overlap in neural activity does not remain confined to the motor execution domain but also occurs, for example, between movement preparation and MI [99, 100]. This equivalence in neural activation between movement preparation/execution and MI/AO is thought to account for the improvements in overt motor performance that are commonly seen in healthy individuals after MI/AO training [94, 101, 102]. For example, in a classical study, Pascual-Leone and colleagues (1995) showed that MI training alone, in comparison to physical practice, also led to significant improvements in the overt performance of a fine motor skill with the hand. Moreover, the same neural effects that were induced by physical practice were also found in the individuals undergoing only MI training. In both conditions, there was an increase in the size and excitability of the cortical motor representation of the trained hand [102]. Importantly, the findings from this study demonstrate not only that MI on its own can activate the cortical motor representation(s) of the involved body part(s) but, more critically, that MI training can lead to adaptive stimulation of such representation(s). This adaptive stimulation, in turn, translates into improved overt motor performance with the involved body part(s).

Overall, the above findings have spurred the infusion of MI and AO into neurorehabilitation, particularly into the context of poststroke motor rehabilitation [103–105]. Essentially, the underlying assumption here is that MI or AO training can* reactivate* and adaptively stimulate potentially spared cortical motor representations in the stroke-affected hemisphere. This, in turn, would contribute to increasing activity in the ipsilesional motor cortex and thereby* rebalance* the interhemispheric interactions between this cortical area and the homologous region in the contralateral, unaffected hemisphere. Collectively, this would contribute to improvement of the individual's motor function [91, 106–108] (see the “*reactivation*” and “*rebalancing*” theories in Section 2.2). Interestingly, studies by our group and others have shown that the aforementioned similarity reported in healthy individuals between overt movement and MI/AO, in terms of both the neural substrate that is activated and the ability of both overt movement and MI/AO to induce improvements in overt motor performance after training, is largely preserved in individuals after hemiparetic stroke [109–113]. Because MI and AO do not rely on overt movement execution to activate and adaptively stimulate cortical motor representations, they represent rather promising alternative motor rehabilitation strategies for individuals that have poor residual function after stroke and that therefore cannot effectively engage with the overt practice of physically demanding motor tasks. In this case, individuals can make use of such strategies to initiate motor recovery, for instance, during the beginning of their rehabilitation, while they gradually build up their physical capacity with progressive cardiorespiratory and muscle strengthening/endurance exercises [77, 78], or simply up to a point where they are able to fully participate in overt task-specific training. Furthermore, from this point onwards, and for those who already have higher levels of residual function at the beginning of their rehabilitation, MI- and/or AO-based strategies might be used as complementary interventions to be combined with overt task-specific exercises in order to potentiate motor gains [114].

Thus, it follows that covert practice-based alternative motor rehabilitation strategies for stroke, in comparison to overt practice-based strategies, are more compatible with the wider spectrum of functional recovery and therefore are likely to benefit a larger group of stroke survivors. Nevertheless, despite being promising, research in this arena is still in its infancy and more studies are needed before sound recommendations regarding the use of covert motor strategies such as MI and AO as motor rehabilitation strategies for stroke can be made to effectively inform clinical practice.

#### 5.1.2. Covert Motor Strategies during Upper Limb Immobilisation

In light of the aforementioned, researchers have started to investigate how the brain mechanisms of MI and AO interact with the effects of upper limb immobilisation.

In one study, Bassolino and colleagues (2013) immobilised the upper limb of healthy participants for 10 consecutive hours. During this time, individuals were instructed to imagine, with their eyes closed, reach-to-grasp movements being performed with their restrained arm and hand, “MI group,” observe the same actions through a computer screen, “AO group,” or watch a nature documentary with no human actions, “control group.” The size and excitability of the cortical motor representation corresponding to the immobilised hand were assessed with TMS one day before and immediately after removal of the immobilisation. In the control group, both the size and excitability of the cortical motor representation of the restrained hand were reduced after immobilisation. This finding is in line with the findings from the other immobilisation studies already described earlier in this paper (see Section 4). In the MI group, similar changes were reported. However, in the AO group, no substantial changes were noted. Here, the excitability of the cortical motor representation of the immobilised hand was higher than in the other two groups after immobilisation, and its size remained similar to what was observed in all the three groups before immobilisation [115].

Regarding the behavioural effects of immobilisation, it was recently suggested that they might be attenuated by MI practice before removal of the restriction. In one study, individuals had their left hand immobilised for 24 consecutive hours. After removal of the immobilisation, participants were assessed on a hand recognition task, where their goal was to identify, as quickly as possible, whether a hand displayed on a computer screen corresponded to a left or right hand. Those who did not practice MI during immobilisation showed slower response times in the task, particularly for left hand stimuli [116]. It is not known, however, if this modulation of task performance by MI practice was mediated by changes at the level of the cortical motor representation corresponding to the immobilised hand. In addition, whether the gains in performance on the hand recognition/reaction task reported in this study extrapolate to the domain of overt motor performance also remains unknown.

In retrospect, the finding from the study by Bassolino et al., that MI training was inefficient in adaptively stimulating the cortical motor representation of the immobilised hand [115], appears to be not only in relative dissonance with the finding from others, who have otherwise reported adaptive neural plasticity of cortical motor representations with MI training (e.g., see [102] in Section 5.1.1), but also in contradiction to Jeannerod's “Simulation Theory,” which predicts adaptive stimulation of cortical motor representations with MI training [92]. A possible explanation for this contradiction, which was indeed acknowledged by Bassolino et al., might be that the effects of MI on the motor system, particularly in terms of stimulating cortical motor representations, are influenced by the “state,” that is, the posture, position, and/or history of mobility, of the body parts corresponding to these representations during the imagination process. For instance, studies have shown that if the current state, in this case the posture, of a body part is congruent with the movements/actions being imagined, the size and excitability of the corresponding cortical motor representation increase to a greater extent than when the state is incongruent [117, 118]. This suggests that the afferent, proprioceptive information coming from the body part(s) involved in the imagined movements/actions may play an important role in modulating the effects of MI on the corresponding cortical motor representation(s). Therefore, according to this perspective, the immobilisation of the upper limb in the study by Bassolino et al., by maintaining the hand both in a relatively antagonistic posture and immobile for several hours, that is, in a state which was rather incongruent with the imagined reach-to-grasp movements/actions, would have compromised the effects of MI on the corresponding cortical motor representation [115]. If this is indeed the case, it would have important implications for the use of MI in the context of motor rehabilitation after stroke, especially in cases of low-functioning upper limb hemiparesis. Here, MI training is generally used with the underlying assumption that it can adaptively stimulate cortical motor representations in the damaged cerebral hemisphere and that this might translate into improved motor function (see Section 5.1.1). However, in this context, the state of the patient's paretic upper limb is sometimes largely incongruent with the movements/actions that are usually imagined during MI training, which basically consists of the mental rehearsal of functional movements of the patient's daily living, such as reaching and grasping (see [103] for a review). Not infrequently, the paretic upper limb is found in a state that is characterised by both, a relative immobility or lack of overt voluntary movement and a spastic posture with varying degrees of sustained flexion at the fingers, wrist, and elbow. Such state, in turn, by providing the brain with reduced and/or incompatible proprioceptive information, could decrease or mask the potential of MI as a rehabilitation strategy. In keeping with this hypothesis, Liepert and colleagues (2012) showed that MI of paretic hand movements increased the excitability of the corresponding cortical motor representation in stroke patients with somatosensory deficits to a much lesser degree than in patients with pure motor hemiparesis [119]. Overall, this seemingly important influence of the current state of the body part(s) on the effects of MI on the corresponding cortical motor representation(s) could explain, at least in part, the relative inconsistency in the results from recent clinical trials of MI training after stroke (see [103, 108] for reviews). Thus, given its theoretical and clinical implications, the interaction between the effects of both MI and upper limb immobilisation on the involved cortical motor representations should be investigated in more detail.

We believe the upper limb immobilisation model provides a compelling neurobehavioural framework for exploring covert motor strategies, such as MI and AO, within the context of poststroke motor rehabilitation. This is because upper limb immobilisation not only mimics the maladaptive neural plasticity patterns that are believed to contribute to the motor deficits shown by an individual after a hemiparetic stroke but, critically, also consists of a disease-free model of compromised brain function. Here, covert motor strategies can be investigated in healthy individuals for their potential to adaptively stimulate cortical motor representations, within a context of “stroke-like” maladaptive neural plasticity, without the influence of lesion-related confounding factors that otherwise are inevitably present in disease-based models. This opportunity, in turn, might greatly contribute to sharpening the mechanistic understanding of these strategies and thereby improve their translation to the clinical context of poststroke motor rehabilitation.

#### 5.1.3. The Use of Adjunctive Therapies and the Opportunity to Enhance Adaptive Neural Plasticity during Upper Limb Immobilisation

Recapitulating on the role of the pattern of activity in the efferent and/or afferent neural signalling pathways targeting and/or coming from a particular body part in shaping the corresponding cortical motor representation (see Section 2.1), a recent study tested in healthy individuals the interaction between peripheral somatosensory stimulation (PSS) and upper limb immobilisation. Complementing their previous findings on the effects of immobilisation on both the excitability of homologous cortical motor representations and the interhemispheric interactions between them, Avanzino et al. (2014) showed that a form of proprioceptive stimulation can largely attenuate the maladaptive neural plasticity that is induced by upper limb immobilisation. Specifically, they found that intermittent vibration of a muscle from the immobilised hand delivered throughout the period of immobilisation was able to prevent large decreases in the excitability of the corresponding cortical motor representation and to abolish both, increases in the excitability of the homologous representation in the opposite hemisphere and changes in the interhemispheric balance between the two homologous cortical motor representations [120].

The findings from this latter study, when taken together with those from previously described studies investigating covert motor strategies during immobilisation, invoke the idea that different interventions might be delivered in combination during upper limb immobilisation in order to maximize prevention of maladaptive/promotion of adaptive neural plasticity in the brain's motor system, much like with the use of adjunctive therapies in association with task-specific exercises during poststroke motor rehabilitation (see Section 3). Here, a potential manipulation could be, for instance, to combine covert motor strategies with brain stimulation (BS) and/or PSS techniques during upper limb immobilisation (Figure 2(b)). It is likely that the effects of such strategies, in terms of adaptively stimulating cortical motor representations, would be strengthened by concomitant BS, delivered to either one or both motor cortices, and/or PSS of the immobilised body part(s). A recent study provides empirical support for this prediction. It was shown that observation of right hand movements combined with sensorimotor electrical stimulation of the median nerve at the level of the right wrist increased the excitability of the cortical motor representation of the involved hand, an effect that was not present when the two interventions were delivered separately. In addition, this modulation outlasted the period of observation-stimulation [121]. It remains to be tested, however, whether a similar manipulation would result in a potentiation of the effect of AO during upper limb immobilisation.

## 6. Conclusion and Perspectives

Advances in our understanding of the neural plasticity that occurs after hemiparetic stroke, both adaptive and maladaptive, have contributed to the formulation of theories of motor recovery after stroke. Such theories identify the processes that are likely to promote motor recovery, for instance, of poststroke upper limb hemiparesis, and that therefore might be targeted by neurorehabilitation efforts. Contemporary motor rehabilitation strategies target these processes, but because they are essentially centered in the overt practice of physically demanding task-specific exercises, they do not easily translate into benefit for stroke survivors sustaining low-functioning upper limb hemiparesis. For these individuals, motor rehabilitation options are currently limited. MI and AO are two examples of covert or cognitive motor strategies that have a great potential to translate into alternative motor rehabilitation strategies for stroke. Because they can adaptively stimulate cortical motor representations in the absence of overt movement, MI- and AO-based training strategies have a greater potential for benefiting the wider spectrum of functional recovery after stroke, including those individuals with poor residual function. In this paper, we provided theoretical and empirical evidence that an upper limb immobilisation-based neural plasticity model, by capitalising on current theories of motor recovery after stroke, the shortage of overt movements, and a disease-free condition of compromised brain function, provides a very attractive neurobehavioural template for exploring these strategies within this context.

The findings from the immobilisation studies reviewed here, particularly those concerning the effects of immobilisation on the interhemispheric interactions between homologous cortical motor representations, have important implications for a particular contemporary motor rehabilitation strategy addressing poststroke upper limb hemiparesis. This strategy is known as constraint-induced movement therapy (CIMT). CIMT falls within one of those two categories of interventions that were presented in this paper (see Section 3). Essentially, this rehabilitation strategy consists of three elements [122]. These are (1) repetitive and progressive task-specific training with the paretic upper limb for several hours a day, usually for a period of 2 weeks; (2) a “transfer package” made of behavioural techniques used to promote transfer of the gains obtained during the treatment period in a research and/or clinical setting to the individual's real-world environment; and (3) constraining use of the paretic upper limb through immobilisation of the contralateral, less-affected limb. This particular therapy has proven to be an effective intervention for improving paretic upper limb function after stroke and is currently recommended as the treatment of choice for those individuals with relatively high levels of residual function [123]. Despite the fact that CIMT has been extensively investigated during the past few years, the mechanisms mediating treatment success with this intervention are still relatively poorly understood (see [124] for a discussion). One influential proposal has been that functional improvements after CIMT are consequential to the massed overt motor practice with the paretic upper limb, which, in turn, contributes to both reversal of the learned nonuse phenomenon and adaptive neural plasticity, that is, increases in the size and/or excitability, of the corresponding cortical motor representations [125]. It follows from this proposal that the relevance of immobilising the less-affected upper limb for promoting motor gains with the more-affected, paretic limb is often underestimated. More commonly, an indirect, relatively trivial role is attributed to the immobilisation element of CIMT, for example, that it serves only as a “constraining” instrument employed simply to encourage use of the paretic upper limb. As Taub and colleagues (1999) commented on the relevance of the immobilisation element of CIMT, “There is thus nothing talismanic about use of a sling or other constraining device on the less-affected limb. The common factor appears to be repeatedly practicing use of the paretic arm. Any technique that induces a patient to use an affected limb many hours a day for a period of consecutive days should be therapeutically efficacious. This factor is likely to produce the use-dependent cortical reorganization found to result from CI Therapy (23, 58, 59) and is presumed to be the basis for the long-term increase in the amount of use of the more-affected limb” [126]. As already pointed out by others (e.g., see [83]), recent findings from upper limb immobilisation studies suggest, however, that a more sensible appreciation of the contribution of the immobilisation element of CIMT would be that such manipulation might otherwise have a more direct, meaningful role in mediating treatment success, at least in theory. For example, it is possible that, at least in some individuals, the effects of immobilisation of the less-affected upper limb during CIMT would be superimposed to those effects induced by the intensive motor training with the paretic upper limb. More specifically, as long as a condition of true immobilisation, that is, of substantially reduced usage, of the less-affected upper limb is present, it would decrease activity of the contralesional motor cortex, which, in turn, could contribute to reducing transcallosal inhibition from this region towards the homologous area in the affected hemisphere. As a consequence, this could attenuate interhemispheric imbalance and facilitate ipsilesional activation and hence boost massed practice-induced adaptive neural plasticity (see “*rebalancing*” theory in Section 2.2). Since its original conceptualisation, CIMT has taken many different forms, each of which containing different combinations and/or durations of the three elements of the intervention, which are task-specific training, “transfer package,” and immobilisation of the contralateral, less-affected upper limb [127–129] (see [124] for a review). When considering whether or not to include the immobilisation element of CIMT in the rehabilitation program, the above-mentioned consideration should be taken into account, but also the pros and cons of restraining movements of the patient's less-affected upper limb should be carefully weighted.

On a different note, with the advent of noninvasive brain stimulation (BS) techniques during the past years, it has become possible to develop neural plasticity “protocols” that are able to induce relatively predictable changes in the excitability of selected brain regions and that therefore might be used to improve the individual's behavioural capacity under certain conditions. The motor cortex, in particular, has been a major target of such protocols. Here, BS has been used to either directly induce adaptive neural plasticity of cortical motor representations in healthy individuals with excitatory stimulation and thereby improve their motor learning or adaptively modulate neural plasticity patterns after hemiparetic stroke with excitatory and/or inhibitory stimulation and thereby improve the individual's motor recovery (see Section 3 and also [66, 130] for reviews). More recently, neural plasticity research has shown that it is possible to modulate the response of the motor cortex to neural plasticity protocols, to both those exogenously induced through BS and endogenously induced protocols, such as motor learning, by manipulating its activity before delivery of such protocols [131]. In other words, the strength of an excitatory or inhibitory neural plasticity protocol that targets the motor cortex might be either increased or decreased depending on the history of activity of this area. This priming effect has been termed “metaplasticity” [132]. Basically, if activity in a brain region or neuronal network is high, then the strength of a subsequent excitatory protocol is decreased, while the opposite is true for a subsequent inhibitory protocol. Conversely, if activity in that brain region or neuronal network is low, then a subsequent excitatory protocol is enhanced, while the opposite is true for a subsequent inhibitory protocol. An example of this phenomenon was provided by Jung and Ziemann (2009). Briefly, the authors showed that, in healthy participants, motor learning—an endogenously induced excitatory neural plasticity protocol that targets the motor cortex—can be potentiated by decreasing activity/excitability of the targeted motor cortex with a long-term depression- (LTD-) inducing paired associative stimulation (PAS) protocol delivered before the learning paradigm [133]. Motor learning is thought to improve the individual's motor capacity by, among other processes, inducing long-term potentiation (LTP) and thereby adaptive neural plasticity in their motor cortex (see Section 2.1). In the study by Jung and Ziemann, it is likely that the LTD-inducing PAS protocol delivered before motor practice increased, via metaplasticity mechanisms, the likelihood for subsequent LTP in the involved motor cortex and cortical motor representation(s), hence potentiating motor learning [133]. Recently, this finding has prompted others to extrapolate the concept of metaplasticity to the context of motor rehabilitation after stroke. Interestingly, in a proof-of-principle study, challenging the standard approach in BS-based poststroke motor rehabilitation (e.g., see Section 3 and Figure 1(b)), Di Lazzaro and colleagues (2013) found that, in general, inhibitory, activity-decreasing BS of the ipsilesional motor cortex followed by physiotherapy exercises improved the motor outcomes of patients more than sham BS [134]. The results from these two latter studies, when taken together with the findings reported in this paper concerning the effects of upper limb immobilisation on the motor cortex, might spark rather provocative speculations. As demonstrated by Rosenkranz et al. (2014) (see end of Section 4), apart from decreasing the excitability of the corresponding cortical motor representations in the contralateral motor cortex, upper limb immobilisation also seems to alter neural plasticity mechanisms within these representations, such that the larger the decrease in their excitability induced by the restriction, the greater their sensitivity to subsequent LTP-like processes [86]. This suggests that, like BS, but with the advantages of being much cheaper and technically simpler, upper limb immobilisation could also be used as a noninvasive neural plasticity protocol to decrease motor cortex activity/excitability and, within a context of metaplasticity, improve subsequent motor training. For instance, it can be speculated that, in healthy individuals, immobilisation of the upper limb for a certain period of time which is sufficient to substantially decrease the excitability of the corresponding cortical motor representations in the contralateral motor cortex could perhaps facilitate/enhance subsequent motor learning with that limb by increasing the likelihood for LTP in the involved representations. In the same vein, now within the context of poststroke motor rehabilitation, it can also be speculated that a period of immobilisation of the patient's paretic upper limb before a physiotherapy session could boost subsequent practice-induced adaptive neural plasticity of the corresponding cortical motor representations. This, in turn, could eventually enhance rehabilitation outcomes. Despite their rather provocative and speculative nature, the findings discussed previously suggest that such predictions are, at least theoretically, valid. Future studies could test these hypotheses.

Before upper limb immobilisation can be consolidated as a neural plasticity model/protocol in humans, some issues remain to be addressed by future research. First, most of the studies, if not all, on upper limb immobilisation have been conducted with young participants. It would be important to also perform these investigations with aged populations, especially within the context of translational neurorehabilitation research, as the vast majority of stroke survivors are elderly. Second, there is no consensus yet on a possibly ideal immobilisation time to induce the reported changes in the motor system, both those that are measured at the level of the motor cortex and those expressed behaviourally. For instance, studies have used restriction periods ranging from hours, for example, 6, 8, 10, 12, and 24 hours, to days in humans and to months in animals. Determining the optimal length of immobilisation is critically important to minimise not only costs, but also potential ethical issues that might be present, particularly in long-term immobilisation studies. In this case, the question of whether the effects of immobilisation on the brain's motor system can be potentiated, and perhaps accelerated with exogenous manipulations, such as noninvasive BS, should also be given consideration.
